# Simple Epidemiological Dynamics Explain Phylogenetic Clustering of HIV from Patients with Recent Infection

**DOI:** 10.1371/journal.pcbi.1002552

**Published:** 2012-06-28

**Authors:** Erik M. Volz, James S. Koopman, Melissa J. Ward, Andrew Leigh Brown, Simon D. W. Frost

**Affiliations:** 1 Department of Epidemiology, University of Michigan, Ann Arbor, Michigan, United States of America; 2 Institute of Evolutionary Biology, School of Biological Sciences, University of Edinburgh, Edinburgh, United Kingdom; 3 Department of Veterinary Medicine, University of Cambridge, Cambridge, United Kingdom; Imperial College London, United Kingdom

## Abstract

Phylogenies of highly genetically variable viruses such as HIV-1 are potentially informative of epidemiological dynamics. Several studies have demonstrated the presence of clusters of highly related HIV-1 sequences, particularly among recently HIV-infected individuals, which have been used to argue for a high transmission rate during acute infection. Using a large set of HIV-1 subtype B pol sequences collected from men who have sex with men, we demonstrate that virus from recent infections tend to be phylogenetically clustered at a greater rate than virus from patients with chronic infection (‘excess clustering’) and also tend to cluster with other recent HIV infections rather than chronic, established infections (‘excess co-clustering’), consistent with previous reports. To determine the role that a higher infectivity during acute infection may play in excess clustering and co-clustering, we developed a simple model of HIV infection that incorporates an early period of intensified transmission, and explicitly considers the dynamics of phylogenetic clusters alongside the dynamics of acute and chronic infected cases. We explored the potential for clustering statistics to be used for inference of acute stage transmission rates and found that no single statistic explains very much variance in parameters controlling acute stage transmission rates. We demonstrate that high transmission rates during the acute stage is not the main cause of excess clustering of virus from patients with early/acute infection compared to chronic infection, which may simply reflect the shorter time since transmission in acute infection. Higher transmission during acute infection can result in excess co-clustering of sequences, while the extent of clustering observed is most sensitive to the fraction of infections sampled.

## Author Summary

Diversity of viral genetic sequences depends on epidemiological mechanisms and dynamics, however the exact mechanisms responsible for patterns observed in phylogenies of HIV remain poorly understood. We observe that virus taken from patients with early/acute HIV infection are more likely to be closely related. By developing a mathematical model of HIV transmission, we show how these and other patterns arise as a simple consequence of intensified transmission during the early/acute stage of HIV infection, however observing these patterns is highly dependent on sampling a significant fraction of prevalent infections.

## Introduction

Phylogenetic clusters of closely related virus such as HIV arise from the epidemiological dynamics and transmission by infected hosts. If virus is phylogenetically clustered, it is an indication that the hosts are connected by a short chain of transmissions [1].

If super-infection is rare, and assuming an extreme bottleneck at the point of transmission, each lineage in a phylogenetic tree corresponds to a single infected individual with its own unique viral population [2], [3]. A transmission event between hosts causes an extreme bottleneck in the population of virus in the new hosts. For infections between MSM, it is estimated that infection is initiated by one or several virions [4], [5]. At the time of transmission, the quasispecies of virus within the transmitting host diverges and can thereby generate a new branch in the phylogeny of consensus viral isolates from infected individuals [6]. Transmissions in the recent past should be reflected by recently diverged lineages, and transmissions from long ago should reflect branches close to the root of a tree. [7]. Viruses such as HIV which have a high mutation rate relative to epidemiological spread can generate epidemics such that the correspondence between transmission and phylogenetic branching is most clear [2].

Given a phylogeny of virus reconstructed from 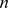 samples, the phylogenetic clusters are a partition of the 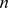 sample units into disjoint sets as a function of the tree topology. A cluster will consist of all taxa of the tree that are descended from a given lineage on the interior of the tree. There are many variations of this idea, and there is no general agreement about how to choose interior lineages for defining clusters. The most common algorithms require strong statistical support for a monophyletic clade among all taxa in a cluster [8]–[14]. These definitions may additionally require all taxa in a cluster to be connected by short branches with less than a threshold length [11], or similarly require that the genetic sequences corresponding to each taxon be separated by a genetic distance less than a given threshold [8], [14]. Definitions of clustering based on statistical support for monophyly are very difficult to operationalize in a mathematical model, and in particular, it is not clear how the statistical significance of internal nodes relates to population dynamics. Consequently, we have devised a conceptually similar definition of clusters that relies on the estimated time to most recent common ancestor (TMRCA) of a set of taxa [15]. A formal definition is provided below.

The sizes of the groupings that arise from a clustering algorithm have been interpreted as a reflection of the heterogeneity of epidemiological transmission. The distribution of cluster sizes of HIV is often skewed right, and depending on the definition of clustering used, can have a heavy tail [14], [15]. This is consistent with the prevailing view among modelers of sexually transmitted infections that there is a skewed and in some cases power-law distribution in the number of risky sexual contacts in the population, however it is not straightforward to make inferences about sexual network properties from cluster size distributions [16]. In the case of HIV, the distribution of branch lengths within clusters may also reflect the disproportionate impact of early and acute HIV infection on forward transmission, which is due to higher viral loads in the early stages of infection, higher transmissibility per act [17], and fluctuating risk behavior [18].

When the taxa of the phylogeny are labeled, such as with the demographic, behavioral or clinical attributes of the the individuals from whom the virus was sampled, one can further analyze statistical properties of clustered taxa. Similar taxa, such as those arising from acute infections, may cluster together (or *co-cluster*) at greater rates. Patterns of co-clustering might be informative about the fraction of transmissions that occur at different stages of infection or between different demographic categories. HIV phylogenies from men who have sex with men (MSM) have been widely observed [12], [13], [19] to have individuals with early/acute HIV infection that are much more likely to appear in a phylogenetic cluster. And moreover, if early-stage individuals are in a cluster, they are much more likely to be clustered with other early infections. Both Lewis et al. and Brenner et al. [8], [9] have hypothesized that co-clustering of early infection is caused by higher transmissibility per act during early infection. For example, in phylogenies with time-scaled branch lengths, if a large fraction of clusters have a maximum branch length of six months [8], [15], this suggests that *at least* that fraction of transmissions also occur within six months. In this article we demonstrate that the mechanisms that generate co-clustering of early infections are complex, and involve many attributes of the epidemic in addition to higher transmissibility per act [17]. To summarize, several features of the phylogenetic structure of HIV in MSM have been independently observed by several investigators:

- Many more early infections are phylogenetically clustered than late infections. For future reference, we will refer to this as *excess clustering* of early/acute infections.
- If an early infection is clustered, it is more likely to be co-clustered with another early infection than expected by chance alone. For future reference, we will refer to this as *excess co-clustering* of early/acute infections.
- The distribution of phylogenetic cluster sizes is skewed to the right and is potentially heavy-tailed.

Below, we illustrate these clustering patterns using 1235 HIV-1 subtype B *pol* sequences collected between 2004 and 2010 in Detroit, Michigan, USA.

These common clustering features motivate several questions. How informative are clustering patters about the underlying epidemic? In particular, how does higher transmissibility per act during early infection shape the phylogeny of virus ? To address these questions, we have developed a simple mathematical framework that demonstrates the connection between epidemiological dynamics and the expected patterns of clustering from a transmission tree and the corresponding phylogeny.

Our modeling work suggests that common features of HIV phylogenies are not coincidences, but universal features of certain viral phylogenies. We expect to see similar patterns for any disease such that the natural history features an early period of intensified transmission. High transmission rates during early infection may be a consequence of higher transmissibility per act due to high viral loads, but are also influenced by behavioral factors, such as fluctuating risk behavior [18], concurrency [20], and a lack of awareness of the infection. We do not explicitly model immunological or behavioral factors, but rather consider a compound parameter that describes the rate of transmission during the early/acute period. We find that while higher transmission rates increase the frequency of early/acute clustering, virus collected from early/acute patients clusters at a higher rate even when transmission rates are uniform over the infectious period.

## Materials and Methods

### Ethics statement

This research was reviewed by the Institutional Review Board at the University of Michigan. Data used in this research was originally collected for HIV surveillance purposes. Data were anonymized by staff at the Michigan Department of Community Health before being provided to investigators. Because this research falls under the original mandate for HIV surveillance, it was not classified as human subjects research.

### Phylogenetic clustering of Michigan HIV-1 sequences

Our analysis consists of an empirical component which establishes clustering patterns for a geographically and temporally delineated set of HIV sequences, and an analytical component which establishes a possible mechanism that could generate the observed patterns.

We examined the phylogenetic relationships of 1235 HIV-1 subtype B partial-*pol* sequences originally collected for drug-resistance testing. All sequences were collected in the Detroit metropolitan statistical area between 2004 and 2010. Sequences were tested for quality and subtype using the LANL quality control tool [21]–[23], and aligned against a subtype-B reference (HXB2).Drug resistance sites [24] were treated as missing data.

A maximum clade credibility phylogeny was estimated with BEAST 1.6.2 [25]. The phylogeny was estimated using a relaxed molecular clock and and HKY85 model of nucleotide substitution with Gamma rate variation between sites (4 categories). The MCMC was run for 50 million iterations with sampling every 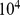 iterations. The first million iterations were discarded. The effective sample size of all parameters exceeded 50.

The phylogeny was converted into a matrix of pairwise distances between taxa expressed in units of calendar time. The distance between a pair of taxa was the TMRCA estimated by BEAST. Taxa were then classified into clusters using hierarchical clustering algorithms. A pair of taxa were considered to be clustered if the estimated TMRCA did not exceed a given threshold, and a range of thresholds was examined, from 0.5% of the maximum distance to the distance corresponding to the point where 90% of taxa are clustered with at least one other taxon.

Co-clustering of early/acute infections was investigated using a clinical variable (CD4 count) and a measure of genetic diversity of the virus. Both CD4 and sequence diversity are imprecise indicators of stage of infection. Nevertheless, with a large population-based sample, even noisy indicators of stage of infection are useful for illustrating phylodynamic patterns.

In most cases, CD4 counts were assessed contemporaneously with samples collected for sequencing. The CD4 cell counts can be informative about disease progression and can be used as a noisy predictor of the unknown date of infection [26]. Individuals with very high cell counts are unlikely to represent late/chronic infections, and we hypothesize that virus from these patients will be more likely to be phylogenetically clustered. Clustering of patients with high CD4 was previously observed by Pao et al. [10]

Recent work [27] has also highlighted the potential for sequence diversity to be informative of the date of infection. The frequency of ambiguous sites (FAS) in consensus sequences provides an approximate measure of sequence diversity in the host. HIV infection is initiated by one or a few founder lineages [4], [5]; initially the diversity of the viral population within the host is low, but diversity increases steadily over the course of infection [28]. By convention, consensus sequences report ambiguous sites as those where the most frequent nucleotide is read with a frequency less than 80%. We hypothesize that having few ambiguous sites is an indicator of early/acute infection; sequences with fewer ambiguous sites will be more likely to be in a phylogenetic cluster and to be clustered with other sequences with few ambiguous sites.

A simple analysis was conducted to establish the existence of excess clustering and co-clustering in the Michigan sequences. This analysis is not designed to classify our sample into a early/acute component or to estimate the date of infection for each unit.

To illustrate excess clustering of early/acute infections, we calculated the mean CD4 cell count and FAS for each sample unit in a phylogenetic cluster. Because all clustering thresholds are arbitrary, we explored a large range of values, up to the point where 90% of the sample was clustered with at least one other unit. The standard error of the estimated mean was calculated assuming simple random sampling. For small threshold distances, very few taxa are clustered, and the standard error is large, but decreases monotonically as the threshold is increased and more taxa are clustered.

To illustrate excess co-clustering, we classified taxa into three categories of CD4: those with CD4 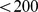, representing AIDS cases; those with CD4 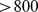, and those with CD4 between 200 and 800. Taxa were also classified into quartiles by FAS. We then counted the number of pairwise clusterings of taxa within and between each category. These counts were arranged in a matrix. Large counts along the diagonal (within categories) represent co-clustering by stage of infection. To establish excess co-clustering, we compared the counts to the expectation if clusters were being formed at random, e.g. if two taxa were selected uniformly at random without replacement.We denote the symmetric matrix of co-clustering counts as 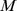, so that 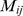 represents the number of times that a taxon in category 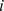 is clustered with a taxon in category 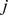. The sum of counts in the 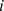
'th row of 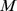 will be denoted 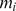. Following the methods described in [29], the expected value of 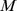 under random pair formation is
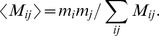
Below, we illustrate the difference 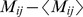. We can also calculate the assortativity coefficient [29], 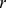, which describes the total amount of co-clustering in the matrix. To construct the co-clustering matrices, we selected the value of the distance threshold which maximized the assortativity coefficient.

### Mathematical model

Following the approach outlined in [6] and [30], we develop a deterministic coalescent model derived from a compartmental susceptible-infected-recovered (SIR) model. A system of several ordinary differential equations describe the dynamics of prevalence of early and late HIV infection. Individuals pass from a susceptible state, to an early/acute infection state, to a chronic infection state followed by removal (treatment or death). 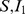, and 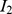 will denote the numbers susceptible, acute, and chronically infected respectively, and the population size will be denoted 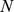. For didactic purposes, we will suppose that treatment is completely effective at preventing forward transmissions. The HIV model is described by the following equations:
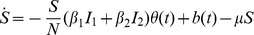


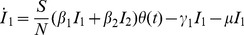
(1)

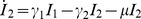
In these equations, 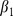 and 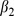 are respectively the frequency-dependent transmission rates for early and chronic infected individuals. The average duration of early and chronic infection are respectively 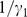 and 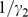. Natural mortality occurs at the rate 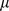 and immigration into the susceptible state occurs at the rate 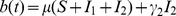, which maintains a constant population size 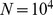. 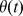 is a term which modulates the way incidence of infection scales with prevalence. For the results presented below, we choose 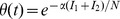. This term corrects for observed patterns of decreasing incidence with prevalence; this can occur as a result of population heterogeneities (including sexual network structure) or as the result of decreasing risk behavior as knowledge of the epidemic spread. Many more relevant details could be included in a model of the HIV epidemic in MSM, however our purpose is to demonstrate how these simple dynamics lead to observed phylogenetic patterns.

In [6], a similar HIV model was presented along with a method to fit such models to a sequence of phylogenetic divergence times (the heights of nodes in a time-scaled phylogeny). Where possible, we will use the parameter estimates from [6]. The parameters are reported in table 1. Together, these parameters imply 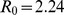 and that 41% of transmissions occur during the acute stage.

**Table 1:** Epidemiological parameters.

Parameter	Symbol	Value
Transmission rate of early/acute	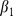	1 per 47 days
Transmission rate of chronic	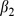	1 per 1207 days
Mean duration of risk behavior	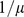	19.5 years
Mean duration of early/acute period	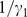	180 days
Mean duration of chronic period	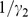	10 years

Corresponding to an epidemic model of the form 1, we can define a coalescent process [31], [32] that describes the properties of the transmission tree and by extension the phylogeny of virus. The taxa descended from a lineage at time 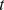 in the past form a clade, which we will also call a *cluster*. The number of taxa in a randomly selected cluster will be a random variable. The *cluster size distribution* (CSD) is a function of a threshold TMRCA 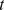, and describes the probability of having a size 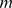 cluster if a lineage (i.e. branch) at time 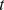 is selected uniformly at random from the set of all lineages at 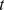 and the size of the cluster descended from that branch is counted. A schematic of how clusters and the CSD are constructed given a tree and a threshold is shown in figure S5. In [6] we derived differential equations that describe the moments of the CSD.

Some of the properties of phylogenies that we seek to reproduce with the model developed below are:

- The number of lineages as a function of time (NLFT), also known as the *ancestor function*.
- The fraction of sampled early/acute and chronic infections which are clustered given a threshold TMRCA.
- Within a given cluster there will a number of early/acute taxa and a number of chronic taxa. We will calculate the correlation coefficient between these counts across all clusters given a threshold TMRCA.
- The moments of the distribution of cluster sizes, including the mean, variance, and skew of cluster sizes.

**Figure 1:**
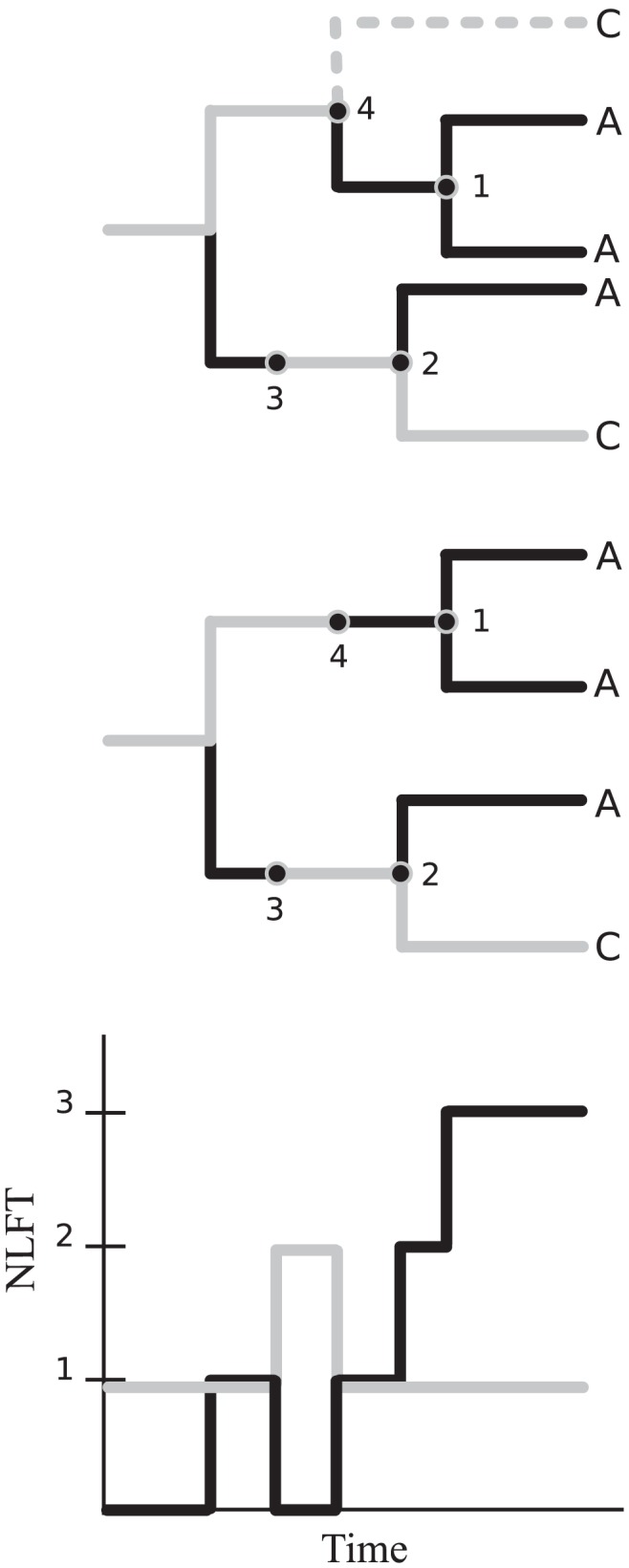
A simple gene genealogy that could be generated by the HIV model. Dark branches with taxa labeled *A* correspond to stage-1 (early/acute infected hosts). Light branches with taxa labeled *C* correspond to stage-2 (chronic infections). Event 1 represents the coalescence of two lineages corresponding to early/acute infection. Event 2 represents coalescence of an early and a late infection. Event 3 represents the stage transition of an early infection to a late infection. Event 4 represents the transmission by a late infection which is not ancestral to the sample. Top: Includes an unsampled lineage (dashed). Middle: The unsampled lineage has been pruned from the tree. The point where the lineage is pruned corresponds to event 4. Bottom: The number of lineages as a function of time (NLFT) which correspond to a host with early/acute infection (black) or chronic infection (grey).

Figure 1 shows a simple genealogy that could be generated by the HIV model. Four events can occur in this genealogy representing coalescence or the changing stage of a lineage. By quantifying the rate that these events occur using a coalescent model, we can calculate the clustering properties of these genealogies. These methods are described below and in detail in supporting Text S1.

The ancestor function is strictly decreasing in reverse time and converges to one (a single lineage) when the most recent common ancestor of the sample is reached. The initial value of the ancestor function (when the population is sampled) is equal to the sample size 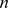. For the purposes of modeling phylogenetic properties of HIV, we will be interested in phylogenies such that the taxa are labeled with the state of the sampled individual (e.g. the individual will have early or late infection corresponding to the states in equation 1). In this case, we will have two ancestor functions, since a lineage may correspond to an infected individual with either early or late infection.

The ancestor functions derived from equations 1, and which are derived in the Text S1 are as follows:
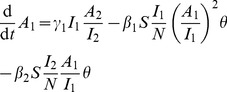
(2)

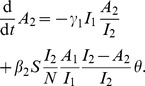
In these equations, 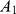 is the number of lineages corresponding to early infections and 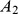 is the number of lineages corresponding to late infections. These equations provide a deterministic approximation to the NLFT, which is 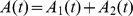. Each term in these equations accounts for loss or gain of lineages due to the concurrent processes of transmission (at rates 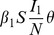 and 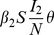) and transition between states (at rates 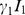). This approximation becomes exact in the limit of large sample and population size. Note that since the model is continuous in both time and state variables, the ancestor functions are not integers in contrast to most coalescent frameworks based on discrete mathematics.

Real epidemics in a finite population will have transmission trees such that the number of lineages at any time is a random variable. The mean-field model presented in equation 1 can be viewed as a description of the dynamics of a stochastic system in the limit of large population size. In this case, we can adapt the coalescent to make approximate descriptions of the stochastic properties of the transmission tree in large populations. The ancestor functions will reflect the approximation of the actual (random) number of lineages. Previous work has demonstrated that deterministic descriptions can be excellent approximations for the number of lineages over time [6], [33]. In the following section, we compare our deterministic coalescent to stochastic simulations, confirming that it is a good approximation over a wide range of parameters.

Given a clustering threshold TMRCA 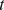, the random variable 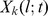 will be the number of stage-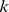 taxa descended from a given lineage 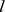 that is extant at time 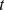 in the past. As before, 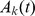 will be the number of type 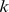 lineages at the time 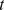 in the past. In our model, infected can be of two types (early/acute and chronic infected), so there are only two types: 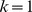 corresponds to earl/acute and 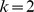 corresponds to chronic. We will denote the set of all lineages of type 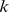 at time 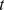 in the past as 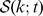. Then we define the 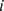 and 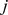
'th moment of cluster sizes descended from a type 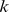 lineage to be
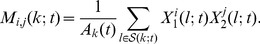
(3)


Many summary statistics that are potentially informative about transmission dynamics can be derived from these moments. The moments are difficult to interpret, so in practice we use them to calculate summary statistics such as variance and skew of the CSD. Below, we examine 30 summary statistics derived from the first three moments and multiple clustering thresholds.

For example, the variance of cluster sizes counting only type 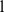 taxa descended from type 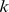 lineages is
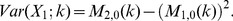
(4)The total variance of cluster sizes counting only stage 1 taxa is found with the weighted average over lineage types:
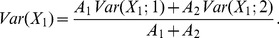
(5)A similar set of equations can be developed for the cluster sizes aggregated over taxon types, that is, for 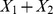. Detailed derivations are provided in Text S1 for differential equations that describe these moments as function of the threshold 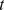.

Event-driven stochastic simulations were conducted to verify the suitability of the deterministic approximations for inference. Simulations implemented a variation on the Gillespie algorithm [34]. Populations consisted of 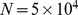 agents, and were simulated for 15 or 30 years starting with one hundred initial infections. At the end of each simulation, a sample of either 20% or 100% of prevalent infections was taken and used to reconstruct a transmission tree. Five hundred simulations were conducted for each sample fraction and sample time. Corresponding to each simulation, 10 transmission trees were generated based on a random sampling of using distinct clustering thresholds. The CSDs were then estimated from each tree and the moments of these distributions were compared to the moment equations (3–5).

We have further conducted an investigation into the potential of various summary statistics of the viral phylogeny for inference of underlying epidemiological parameters. Of particular interest is the fraction of transmissions that occur during early HIV infection. As indicated above, it is possible that phylogenetic clustering of early infections reflects elevated transmission during early/acute HIV infection, which we will define as the infectious period from zero to six months. The following simulation experiment was carried out to identify informative statistics:

1. Parameters 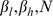 were sampled from a multivariate uniform distribution. 1800 replicates were sampled.
2. For each set of parameters, the HIV ODE model was integrated. The number of transmissions by early/acute and chronic cases was recorded. The number of stage transitions from acute to chronic was also recorded.
3. For each record of transmissions and stage transitions, a coalescent tree was simulated using the method described in [35].
4. For each coalescent tree, summary statistics were calculated and recorded. These statistics consisted of the following: The number of lineages as a function of time before the most recent sample; the correlation between between the number of early/acute and chronic infections with threshold TMRCA; the fraction of acute/recent taxa which remain unclustered (not clustered with any other taxa); the fraction of chronic taxa which remain unclustered; the mean number of taxa clustered with a early/acute infection; the mean number of taxa clustered with a chronic infection. Each of these statistics was calculated using 5 threshold TMRCA uniformly distributed between one year and 25 years before the most recent sample.

The coalescent tree was simulated such that the sample size matched that of the Detroit MSM phylogeny, and the heterochronous sampling of that phylogeny was reproduced in the coalescent tree. Furthermore, the number of early/acute versus chronic taxa sampled was determined using the BED test for recency of infection for each patient [36], and simulations were also made to match the numbers of early/acute and chronic taxa sampled. Virus from patients with early/acute infection accounted for 24% of the samples.

Summary statistics were centralized around the mean and rescaled by their standard deviation (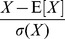). The dependent variable of interest is the fraction of transmissions attributable to the acute stage at the beginning of the epidemic, which may be defined
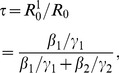
(6)where 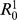 is the expected number of transmissions generated during early/acute infection at the beginning of the epidemic, and 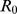 is the expected number of transmissions over the entire infectious period. Pearson correlation coefficients were calculated for each statistic and 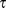. To give a better indication which statistics would be useful for estimating the ratio of acute to chronic transmission rates, we conducted a partial least-squares (PLS) regression [37], which has been used by other investigators when estimating parameters by approximate Bayesian computation (ABC) methods [38]. Prediction error was assessed with 10-fold cross validation. We controlled for the sample fraction by including the prevalence of infection at the time of the most recent sample as a covariate.

## Results

**Figure 2:**
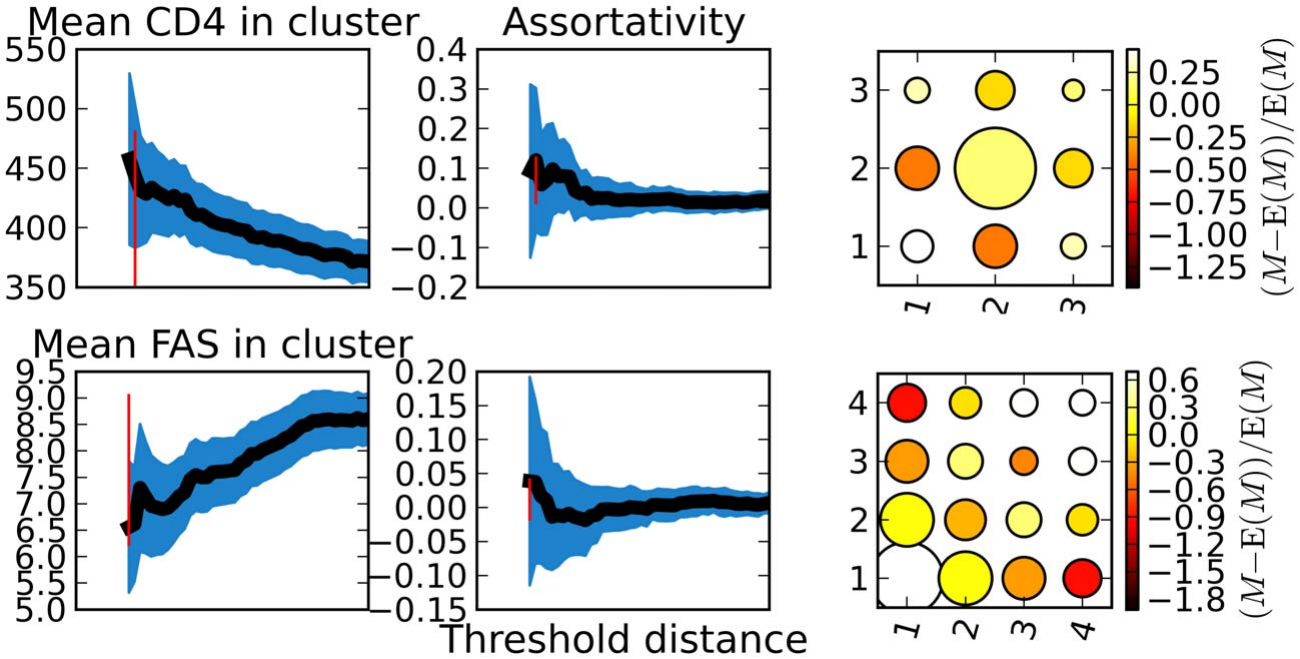
Excess clustering and excess co-clustering of virus from patients with early/acute infections. Left: The mean CD4 cell count (top) and frequency of ambiguous sites (bottom) versus the threshold TMRCA used to form clusters. Middle: The assortativity coefficient, a measure of similarity of co-clustered taxa, versus the treshold TMRCA used to form clusters. Assortativity of CD4 is at top, and frequency of ambiguous sites is bottom. Right: The size of each matrix element is proportional to number of co-clusterings between taxa categorized by CD4 (top, 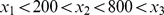) or quartile of frequency of ambiguous sites (bottom). The color represents the extent to which the count of co-clusterings exceeds the expectation if clusters were forming at random. The color scale (far right) shows strong assortativity within quartiles. The vertical red bar represents the threshold which was used to create clusters and the matrix derived from the set of clusters. This threshold corresponds to the maximum of the assortativity coefficient for the derived matrix.

The mean CD4 cell count and FAS for clustered taxa is shown in figure 2. Consistent with our hypotheses, patients with higher CD4 count are more likely to yield phylogenetically clustered virus, and the mean CD4 count among clustered patients has an inverse relationship with the threshold TMRCA for clustering. Also consistent with our hypothesis, patients which yield virus with lower FAS (less diverse virus) are more likely to be phylogenetically clustered, and mean FAS has a positive relationship with the threshold TMRCA for clustering. Patients were strongly co-clustered within quantiles. Maximum assortativity values, which measures the similarity of co-clustered taxa were 13% for CD4 and 4.5% for FAS. The maximum assortativity also occurs at low threshold TMRCA for FAS and CD4 (1700 and 1467 days). Very little clustering is observed between the first and last quantiles.

**Figure 3:**
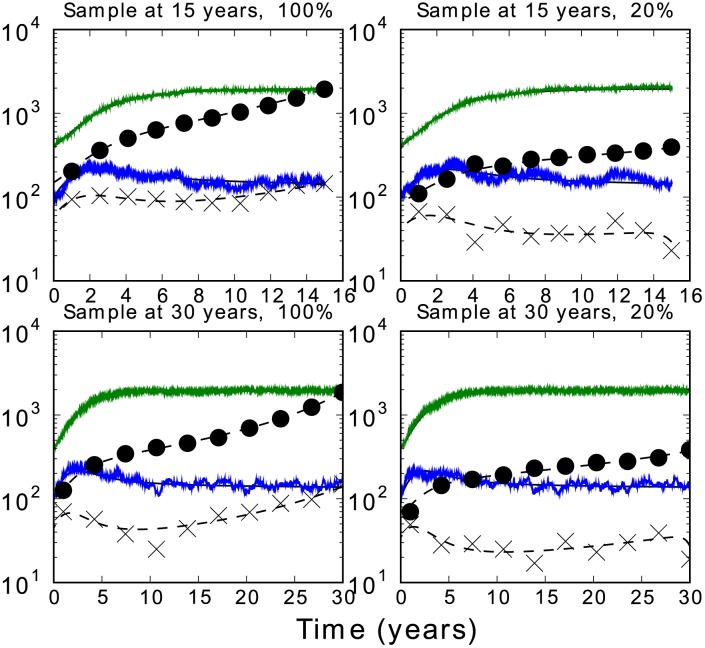
Two simulated epidemics and the deterministic approximations for the prevalent number of early and late infections and the ancestor functions (the number of lineages over time). The x-axis gives the time since the beginning of the epidemic, or equivalently, the threshold TMRCA used to calculate the number of lineages over time. Green describes the simulated number of late infections. Blue describes the simulated number of early infections. Dots show the simulated ancestor function for the number of lineages that correspond to late infections. And x's show the simulated ancestor function for lineages in early infection. Dashed lines show the prediction of the deterministic coalescent. The top row shows results for a sample taken at 15 years following the initial infections, and the bottom shows results for a sample at 30 years. The right column shows results for a sample fractions of 20%, and the left column for a census of prevalent infections(100%).

In general, the deterministic model offers an excellent approximation to the stochastic system. All trajectories pass through or close to the median of simulation predictions. Figure 3 illustrates the prevalence of early/acute and chronic infections from a typical simulation of the HIV model and the corresponding deterministic approximations. This correspondence occurs despite large fluctuations in prevalence when the number of infections is small. In [6] it was shown that the correspondence between the stochastic and deterministic systems can be very good even if the epidemic is started from a single infection and the coalescent is fit to the resulting transmission tree.

In figure 3, late infections outnumber early infections by approximately 20 to 1. As a consequence, NLFT for late infections are more stable due to larger sample sizes, and the NLFT are more noisy for the sample of early infections. The prevalence of infection plateaus prior to the 15 year sample time, so there is not much difference in the phylogenetic features observed at 15 and 30 year sampling times.

**Figure 4:**
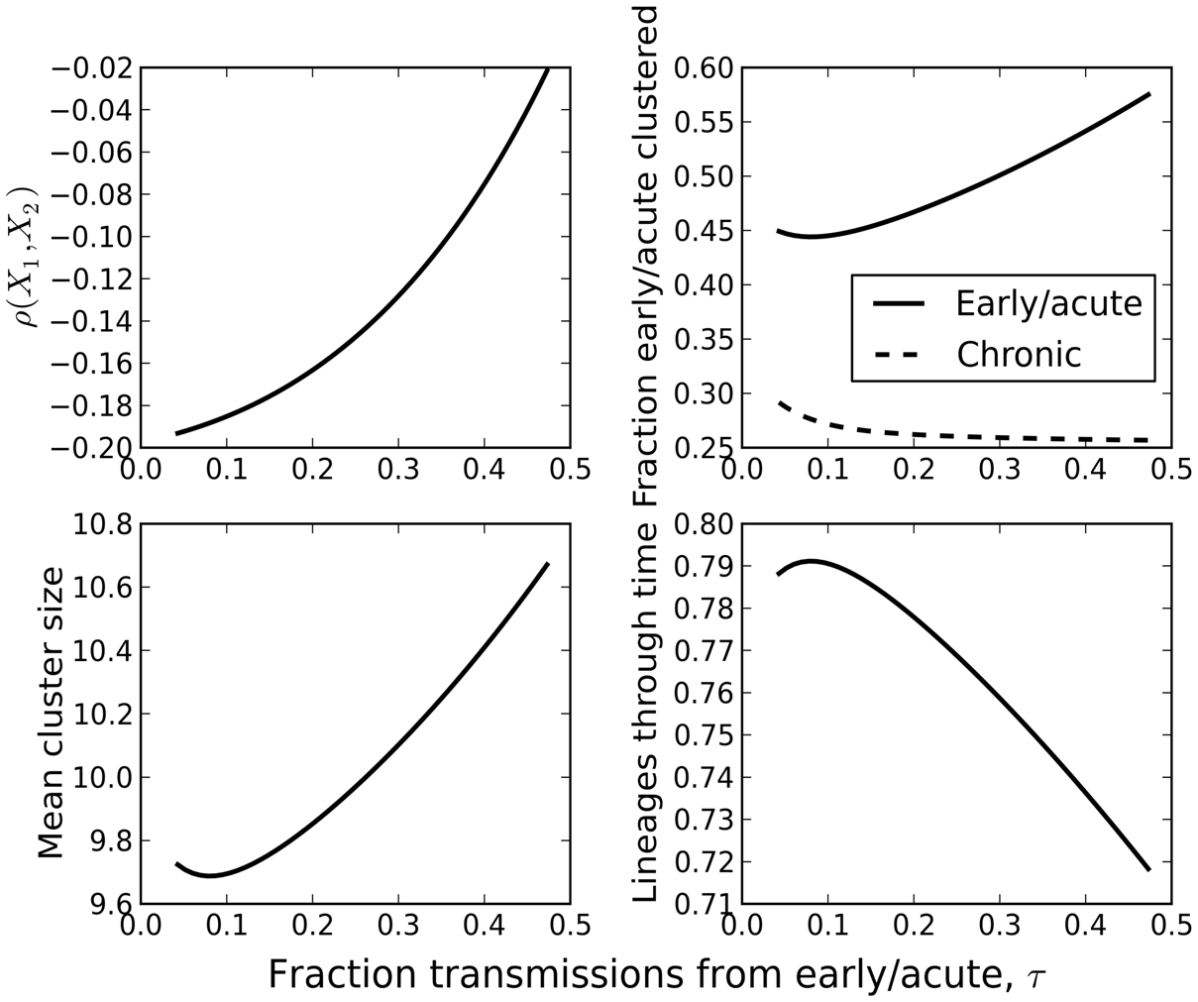
Summary statistics from HIV gene genealogies versus the fraction of transmissions attributable to early/acute infection. The threshold TMRCA was five years before the most recent sample. Sample size and distribution of samples over time was matched to the Detroit MSM phylogeny.

Many summary statistics calculated from an HIV gene genealogy can be informative about the fraction of transmissions attributable to early/acute infection, 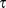 (equation 6). Figure 4 shows the value of four statistics as 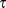 is varied. The dependancy of these summary statistics on the sample fraction is also shown in figure S4. 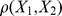 (upper left) is the Pearson correlation coefficient between the number of early/acute taxa and chronic taxa in a cluster and is most sensitive to 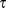. Also shown are the mean cluster size, the number of extant lineages at the threshold TMRCA, and the fraction of taxa in a phylogenetic cluster. As the fraction of transmissions from the early/acute stage is varied, transmission rates 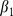 and 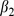 are adjusted so that 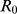 remains constant. The smallest value of 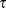 shown in figure 4 corresponds to the point where 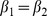, such that there is no excess transmission in the early/acute stage. The most recent sample is assumed to be at 35 years following the initial infection. Epidemic prevalence after 35 years is approximately constant. The threshold TMRCA was five years before the most recent sample. Sample size and distribution of samples over time was matched to the Detroit MSM phylogeny. Furthermore, the number of early/acute versus chronic taxa sampled was made to match the Detroit data by use of the BED test [36] for determining recency of infection.

The fraction of taxa which are phylogenetically clustered also varies with 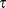 (figure 4, upper left). The fraction of early/acute taxa clustered is more sensitive to 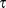 than the fraction from chronic taxa. Early/acute taxa are always clustered at a greater rate than chronic taxa, even when 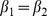 corresponding to the minimum value of 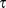. This is because virus from early/acute patients was recently transmitted, making it much more likely that the lineage will coalesce in the recent past regardless of the source of the infection.

Using the mathematical model, we explored many parameters including the threshold TMRCA for clustering, the sample fraction, and the time relative to the beginning of the epidemic at which sampling occurs. Figures S1, S2, S3 demonstrate that the deterministic model is capable of reproducing many phylogenetic signatures that have been associated with HIV epidemics in MSM. For example, figure S5 shows the fraction of the sample (both early and late infections) which remain unclustered with any other sample unit. When the threshold TMRCA is zero (corresponding to the far right of the time axis), the entire sample remains unclustered. As the threshold TMRCA increases (moving leftwards on the time axis), more sample units become clustered and the fraction of taxa remaining unclustered decreases.

The time of sampling makes little absolute difference to the qualitative nature of the tree statistics if sampling occurs after the peak epidemic prevalence (around 15 years). However the sample fraction (the fraction of prevalent infections sampled) has a large effect on all tree statistics. When the sample fraction is large, the fraction remaining unclustered drops much more precipitously than when it is small as the threshold TMRCA increases. This occurs because each transmission can cause a sample unit to become clustered; a large sample size implies that transmissions will have a greater probability of resulting in an observable coalescent event (e.g. it results in a larger ratio 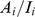).

Early infections become clustered at a much greater rate than late infections. This corresponds to the excess clustering of early/acute infections observed in many phylogenies. By virtue of being infected in the recent past, an acute infection inevitably has a very recent common ancestor with another infection who transmitted to that individual. Mathematically, this is reflected in transmission terms of the form 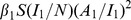 which appear in the ancestor function for early, but not late infections.

When the sample fraction is non-negligible, the fraction of the sample in a cluster levels off for intermediate thresholds. Similar phenomena were noted by Lewis et al. [8] and Hughes et al. [14] who observed that the fraction of the sample in a cluster did not change substantially beyond a small threshold, though these studies probably had high sample fractions. The plateau is due to the bimodality of coalescence times induced by early infection dynamics. Many coalesce events occurs at thresholds close to the sampling time, which corresponds to lineages of early infection coalescing.A larger group of coalescence times occurs close to the beginning of the epidemic when the effective population size is small. We hypothesize that the amount of excess clustering of early infections can be informative for estimating the sample fraction when it is not known.

Figure S2 shows the Pearson correlation coefficient for the number of co-clustered early and chronic infections as a function of the clustering threshold (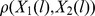). Given that a sample unit is in a cluster, under certain circumstances, it is much more likely to be clustered with another unit of the same type. This is reflected by large negative correlation coefficients for the number of co-clustered early and late infections for small threshold TMRCA. But negative correlation between the number of early and late infections is only observed for small sample fractions and small threshold TMRCA. The region of negative correlation appears very briefly for a 100% sample fraction; the region is much longer for small samples. This implies that if a patient with early infection is clustered, it is much more likely to be clustered with another early infection than expected by chance alone.

The skewness of the CSD shows a similar trend (figure S3). The skewness is always positive (to the right) and rapidly decreases as the threshold TMRCA is increased reflecting greater probability mass in the tail of the distribution. Skew is greatest for small threshold TMRCA, when most clusters are of size 1. The distribution remains positively skewed, though it quickly levels off for intermediate threshold TMRCA. The mathematical model shows that all moments of the CSD are finite and diverge to infinity in the limit of large sample size and threshold TMRCA.

A practical consequence of having an intermediate to large sample fraction is that chains of acute-stage transmission will account for many of the clusters observed at low thresholds. If a taxon is clustered with an early infection, then it is *more* likely that the unit will be clustered with additional early infections since such cases are highly infectious and have likely transmitted in the recent past. This provides a justification for the theory expounded in Lewis et al. [8] that high clustering of cases with recent MRCA's indicates episodic transmission; chains of transmission by early infections are interrupted by occasional long intervals until a transmission by late stage infections.

Corroborating figure 4 which shows that many statistics are correlated with 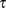, the PLS regression did not single out any particular group of statistics as being informative of early/acute stage transmission rates. The first component distinguishes between statistics that describe co-clustering (correlation of the number of acute and chronic taxa in a cluster) and statistics that describe excess clustering (e.g. the fraction of early/acute taxa that are not clustered with any other taxa). Four principal components were required to explain 42% of the variance of the transmission fraction with additional components only explaining an additional 2%. All statistics were well represented in the model with four components.

## Discussion

We have used coalescent models to characterize the phylogenetic patterns of a virus which produces an early stage of intensified transmission followed by a long period of low infectiousness. These patterns have been observed in multiple phylogenies of HIV-1 from MSM and IDU, and our model suggests that these should be general features for epidemics which feature early and intense transmission. These patterns are not necessarily a consequence of complex sexual network structure [14]. Complex transmission dynamics driven by sexual networks are undoubtedly taking place, but detecting the phylogenetic signature of sexual network structure will require carefully-chosen summary statistics [15]. We have characterized phylogenies using the cluster size distribution (CSD) which is similar to commonly used clustering methods based on strong support for monophyly but is nevertheless tractable for mathematical modeling in a dynamical systems framework. Moments of the CSD reflect a wide range of tree topologies, such as the distribution of branch lengths and tree balance, and are potentially informative of a wide range population genetic processes. For example, a highly unbalanced tree would have produce very skewed CSD, and a very star-like tree would have a CSD that is insensitive to changes in the clustering threshold.

While there has been much discussion of how clustering of acute infections is caused by the intensity of transmission during the acute stage, the amount of excess clustering that will be observed is also very sensitive to the sample fraction. And even if transmission rates in the early/acute stage are equal to those in the late/chronic stage, we would still observe excess clustering of early/acute provided the sample fraction was large enough. This is a simple consequence of early/acute infections being connected by short branch lengths to the individual who transmitted infection. An advantage of the coalescent framework used in this investigation is that it is accurate even with large sample fractions [35].

Some of the statistics which are most informative of the underlying epidemiological processes are those based on co-clustering of labeled taxa, such as the correlation between the number of early and late infections in a cluster. Such statistics tend to be the most responsive to variation of the intensity of transmission during early infection, and are therefore good candidates for future estimation of the fraction of transmissions that occur during the first few months of infection with HIV. Knowing the frequency of early transmission is essential to prevention efforts, since these transmissions are the most difficult to prevent. Individuals with early and acute infection are usually not aware of the infection, and are therefore not susceptible to many interventions. Modeling to evaluate strategies such *seek, test, and treat* (STT) [39], [40] and *pre-exposure prophylaxis*(PrEP) [41] will require good estimates for the frequency of early-stage transmission in diverse populations, and phylogenetic data promise to refine these estimates.

Future work could focus on finding ways to use statistics derived from the CSD for estimation of epidemiological parameters within an approximate Bayesian framework [38], [42], [43]. Alternatively, advances [35] in coalescent theory may make it possible to calculate the likelihood of a gene genealogy conditional on a complex demographic history, such as those generated by the HIV model discussed here. Current techniques are limited in the amount of phylogenetic data that can be used for inference of demographic and epidemiological parameters. Estimation of the intensity of early stage transmission will likely require co-clustering statistics similar to the moments derived from the CSD. In cases where the simple compartmental models fail to reproduce phylogenetic patterns, a more complex transmission system model and its corresponding coalescent should be investigated which might involve sexual networks or geographical [44] and risk heterogeneity. We further conclude that care must be taken in using phylogenetic clusters for epidemiological inference. Mechanisms that generates clustering are often complex and counter-intuitive. We recommend that investigators shift from individual-based inference using small clusters to model-based inference using population-based surveys of sequence diversity.

## Supporting Information

Figure S1 **Two simulated epidemics and the deterministic approximations for the fraction of the sample which remains un-clustered as a function of the threshold TMRCA.** The fraction un-clustered is shown for sample units classified as early infections (solid lines) as well as sample units that are late infections (dashed). The x-axis gives the clustering threshold in units of days since the start of the epidemic. All variables are illustrated for a sample at 30 years following the initial infections and at two possible sample fractions (100% or 20%).
(EPS)

Figure S2 **Simulated epidemics and the deterministic approximations for the Pearson correlation coefficient between the number of co-clustered early and late infections.** Variables are shown as a function of the threshold TMRCA in units of days since the beginning of the epidemic. All of these variables are illustrated for a sample at 30 years following the initial infections and at two possible sample fractions (100% or 20%).
(EPS)

Figure S3 **Two simulated epidemics and the deterministic approximations for the skewness of the cluster size distribution (third central moment divided by the standard deviation cubed).** Variables are shown as a function of the threshold TMRCA in units of days since the beginning of the epidemic. All variables are illustrated for a sample at 30 years following the initial infections and at two possible sample fractions (100% or 20%).
(EPS)

Figure S4 **Summary statistics from HIV gene genealogies versus the fraction of infections sampled after 35 years.** The threshold TMRCA was five years before the most recent sample. Sampling was homochronous.
(EPS)

Figure S5 **Construction of the cluster size distribution (CSD).** Given a tree and a threshold time to most recent common ancestor, represented by red, green, and blue lines, the set of taxa at the base of the tree are classified into disjoint sets or *clusters*. The distribution of cluster sizes for each threshold is shown at right.
(EPS)

Text S1 **Detailed derivations and simulation methods.**
(PDF)
