# Supplementary material for: Simple Epidemiological Dynamics Explain Phylogenetic Clustering of HIV from Patients with Recent Infection
Source: PLoS Comput Biol. 2012 Jun 28;8(6):e1002552. doi: 10.1371/journal.pcbi.1002552 (PMC3386305; doi:10.1371/journal.pcbi.1002552)
Supplement: Text S1 — Detailed derivations and simulation methods. (PDF) [file pcbi.1002552.s006.pdf]

Text S1: Simple epidemiological dynamics explain phylogenetic clustering of HIV from patients with recent infection

Erik M Volz[1,*], James S Koopman[1], Melissa J Ward[2], Andrew Leigh Brown[2], Simon D W Frost[3]
1 Department of Epidemiology, University of Michigan, Ann Arbor, MI, USA
2 Institute of Evolutionary Biology, School of Biological Sciences, University of Edinburgh, UK
3 Department of Veterinary Medicine, University of Cambridge, UK
∗ E-mail: erikvolz@umich.edu

A Derivations of the coalescent model

In [1], [2] and [3], a method for calculating the number of lineages as a function of time (NLFT) was presented for a population genetic model such that the number of infected is described by a nonlinear dynamical system. Recursive equations were also presented for calculating the mean, variance, and all higher moments of the CSD. We briefly review this method and demonstrate how to extend this method to calculate the correlation coefficient between the number of early and chronic infections in a cluster.

The following discussion is adapted from [3]. Consider the equations for the number of stage-1 and stage-2 infected with $\theta(t) = 1$:

$$\begin{aligned}\dot{I_1} &= \frac{S}{N}(\beta_1 I_1 + \beta_2 I_2) - \gamma_1 I_1 - \mu I_1 \\ \dot{I_2} &= \gamma_1 I_1 - \gamma_2 I_2 - \mu I_2\end{aligned}$$

A gene genealogy which might be generated by this HIV model is shown in figure 1. The dark branches correspond to infected hosts in the first stage of infection, and light branches correspond to hosts in the second stage. Moving from the base of the tree to the root (backwards in time), four events can occur, and the coalescent model is specified by the rates that these four events happen:

1. Two dark branches can coalesce, representing transmission by a stage-1 infection.
2. A dark and a light branch can coalesce, representing transmission by a stage-2 infection.
3. A light branch can become dark, representing stage transition (ST) from stage-2 to stage-1 on the reverse time axis.
4. A dark branch can become light, representing transmission by a stage-2 infected that is not ancestral to the sample; these will subsequently be called "invisible transmission" (IT) events.

The last event, IT, is easily forgotten and requires further explanation. When a stage-2 infected transmits and initiates a line of descent that is eventually sampled, but has no other extant progeny in the sample, it will not be manifested in a genealogy as a coalescence. A coalescence would require the transmitting individual to be sampled or to have transmitted to other lineages that are ancestral to the sample. Rather, this event will appear as the branch changing stage from a stage-1 host to the transmitting stage-2 host in reverse time. Two light branches never coalesce since chronic hosts can only generate an acute-stage infection.

Conditional on a transmission by a stage 1 infected, the probability of a coalescence c_1 is that of a conjuction of two events: both the donor and the recipient must be ancestral to the sample. The probability of the former is A_1/I_1 and the probability of the latter given that the donor is ancestral is $(A_1 - 1)/(I_1 - 1)$. Then we have

$$c_1 = \frac{A_1}{I_1}\frac{A_1 - 1}{I_1 - 1} \approx \left(\frac{A_1}{I_1}\right)^2. \tag{1}$$

This approximation is asymptotically exact in large population and sample size.

We can do the same calculation if a stage-2 host transmits infection. The host that is infected must be in the first stage, and the host that transmitted is in the second stage. The probability that the former is ancestral to the sample (i.e. that the host corresponds to a lineage in the tree) is A_1/I_1 and that the latter is ancestral to the sample is A_2/I_2. Then the probability of coalescence is

$$c_2 = \frac{A_1}{I_1} \times \frac{A_2}{I_2}. \tag{2}$$

The probability of the third type of event (ST) is straightforward. Given that a host changes stage, which occurs at the rate $\gamma_1 I_1$, the probability that the host is ancestral to the sample is A_2/I_2.

The probability of observing the fourth type of event (IT) is complex. The probability that a stage-2 host transmits and is not ancestral to the sample is $(I_2 - A_2)/I_2$. The probability that the host which becomes infected is ancestral to the sample is A_1/I_1, so the probability of this event is

$$\frac{A_1}{I_1} \frac{I_2 - A_2}{I_2}.$$

Multiplying the above probabilities by the rates at which each event occurs yields the equations for the NLFT:

$$\begin{aligned} \frac{\mathrm{d}}{\mathrm{d}t} A_1 &= \gamma_1 I_1 \frac{A_2}{I_2} - \beta_1 S \frac{I_1}{N} \left(\frac{A_1}{I_1}\right)^2 \\ &\quad - \beta_2 S \frac{I_2}{N} \frac{A_1}{I_1} \\ \frac{\mathrm{d}}{\mathrm{d}t} A_2 &= -\gamma_1 I_1 \frac{A_2}{I_2} \\ &\quad + \beta_2 S \frac{I_2}{N} \frac{A_1}{I_1} \frac{I_2 - A_2}{I_2}. \end{aligned} \tag{3}$$

Note that in the last term of the equation for A_1, we have combined the contributions from two events: coalescence with probability A_2/I_2 and IT with probability $(I_2 - A_2)/I_2$. The initial conditions of these equations may be based on the number of infected individuals sampled in states 1 and 2, and when integrated, these equations will describe the NLFT.

Calculation of skew and correlations of cluster sizes requires recursive derivation of the first ($M_{1,0}$, second ($M_{2,0}$) and third ($M_{3,0}$) moments of the CSD. This is accomplished by adapting the method in [1] to a model with two stages of infection. It's a bit easier to develop equations for the total number descended from lineages of a given type rather than the mean. So $N_i(k;t)$ will denote the number of stage-i taxa descended from stage-k lineages at time t in the past.

$$N_i(k;t) = \sum_{l \in \mathcal{S}(k;t)} X_i(l).$$

Similarly, to find the second moments, define

$$N_i^{(2)}(k;t) = \sum_{l \in \mathcal{S}(k;t)} X_i^2(l).$$

After solving for the $N's$, the mean is retrieved by:

$$\begin{aligned} M_{1,0}(1) &= N_1(1)/A_1, \quad M_{0,1}(1) = N_2(1)/A_1, \\ M_{1,0}(2) &= N_1(2)/A_2, \quad M_{0,1}(2) = N_2(2)/A_2. \end{aligned}$$

The mean number of stage-i infected (regardless of the state of the ancestral lineage) is found by using the weighted mean; for example, the mean number of stage-1 in a cluster is

$$M_1 = \frac{A_1 M_{1,0}(1) + A_2 M_{1,0}(2)}{A_1 + A_2}.$$

Then the second moments are:

$$\begin{aligned} M_{2,0}(1) &= N_1(1)^{(2)}/A_1, \quad M_{0,2}(1) = N_2(1)^{(2)}/A_1, \\ M_{2,0}(2) &= N_1(2)^{(2)}/A_2, \quad M_{0,2}(2) = N_2(2)^{(2)}/A_2. \end{aligned}$$

The variances are

$$\begin{aligned} \mathrm{Var}(X_1;1) &= M_{2,0}(1) - (M_{1,0}(1))^2, \quad \mathrm{Var}(X_1)(2) = M_{2,0}(2) - (M_{1,0}(2))^2, \\ \mathrm{Var}(X_2;1) &= M_{0,2}(1) - (M_{0,1}(1))^2, \quad \mathrm{Var}(X_2)(2) = M_{0,2}(2) - (M_{0,1}(2))^2. \end{aligned} \tag{4}$$

All of the following moment equations have the same general form. The flux between states is tabulated (e.g. the rate that chronic lineage reverts to early/acute stage going backwards in time). The only difference between each set of equations is the coefficient applied to each flux. To simplify the expressions, we will denote the transmissions per unit time made by stage-i hosts as $F_i = \beta_i \frac{S}{N} I_i$. To summarize:

- Stage 2 goes to 1 at rate $\gamma_1 I_1 \frac{A_2}{I_2}$
- Stage 1 goes to 2 at rate: $F_2 \frac{A_1}{I_1}$

Note that for the variables $N_i(j)$, we don't need to keep track of coalescence between stage 1, because that does not alter the composition of clades descended from lineages in stage 1. We have:

$$\begin{aligned} \frac{\mathrm{d}}{\mathrm{d}t} N_1(1) &= \gamma_1 I_1 \frac{A_2}{I_2} \frac{N_1(2)}{A_2} - F_2 \frac{A_1}{I_1} \frac{N_1(1)}{A_1}, \\ \frac{\mathrm{d}}{\mathrm{d}t} N_2(1) &= \gamma_1 I_1 \frac{A_2}{I_2} \frac{N_2(2)}{A_2} - F_2 \frac{A_1}{I_1} \frac{N_2(1)}{A_1}, \\ \frac{\mathrm{d}}{\mathrm{d}t} N_1(2) &= -\gamma_1 I_1 \frac{A_2}{I_2} \frac{N_1(2)}{A_2} + F_2 \frac{A_1}{I_1} \frac{N_1(1)}{A_1}, \\ \frac{\mathrm{d}}{\mathrm{d}t} N_2(2) &= -\gamma_1 I_1 \frac{A_1}{I_2} \frac{N_2(2)}{A_2} + F_2 \frac{A_1}{I_1} \frac{N_2(1)}{A_1}. \end{aligned} \tag{5}$$

The equations for the second moments are similar to those of the first:

$$\begin{aligned}
\frac{\mathrm{d}}{\mathrm{d}t}N_1(1)^{(2)} &= \gamma_1 I_1 \frac{A_2}{I_2}\frac{N_1(2)^{(2)}}{A_2} \\
&+ F_1\left(\frac{A_1}{I_1}\right)^2 2(M_{1,0}(1))^2 \\
&- F_2\frac{A_1}{I_1}\frac{N_1(1)^{(2)}}{A_1} \\
\frac{\mathrm{d}}{\mathrm{d}t}N_2(1)^{(2)} &= \gamma_1 I_1 \frac{A_2}{I_2}\frac{N_2(2)^{(2)}}{A_2} \\
&+ F_1\left(\frac{A_1}{I_1}\right)^2 2(M_{0,1}(1))^2 \\
&- F_2\frac{A_1}{I_1}\frac{N_2(1)^{(2)}}{A_1} \\
\frac{\mathrm{d}}{\mathrm{d}t}N_1(2)^{(2)} &= -\gamma_1 I_1 \frac{A_2}{I_2}\frac{N_1(2)^{(2)}}{A_2} \\
&+ F_2\frac{A_1}{I_1}\left(\frac{N_1(1)^{(2)}}{A_1} + 2\frac{A_2}{I_2}M_{1,0}(1)M_{1,0}(2)\right) \\
\frac{\mathrm{d}}{\mathrm{d}t}N_2(2)^{(2)} &= -\gamma_1 I_1 \frac{A_2}{I_2}\frac{N_2(2)^{(2)}}{A_2} \\
&+ F_2\frac{A_1}{I_1}\left(\frac{N_2(1)^{(2)}}{A_1} + 2\frac{A_2}{I_2}M_{0,1}(1)M_{0,1}(2)\right)
\end{aligned} \tag{6}$$

The covariance and correlation will be derived from the variables S_i (this should not be confused with the number susceptible). Given a lineage of type i, define

$$S_i = \sum_{l\in\mathcal{S}(i;t)} X_1(l)X_2(l),$$

where $\mathcal{S}(i;t)$ is the set of extant lineages of type i at time t in the past. The dynamics of S_1 and S_2 are described by the following equations:

$$\begin{aligned}
\frac{\mathrm{d}}{\mathrm{d}t}S_1 &= \gamma_1 I_1\frac{A_2}{I_2}\frac{S_2}{A_2} + F_1\left(\frac{A_1}{I_1}\right)^2(2M_{1,0}(1)M_{0,1}(1)) - F_2\frac{A_1}{I_1}\frac{S_1}{A_1}, \\
\frac{\mathrm{d}}{\mathrm{d}t}S_2 &= -\gamma_1 I_1\frac{A_2}{I_2}\frac{S_2}{A_2} + F_2\frac{A_1}{I_1}\frac{I_2-A_2}{I_2}\frac{S_1}{A_1} + F_2\frac{A_1}{I_1}\frac{A_2}{I_2}\left(\frac{S_1}{I_1} + M_{1,0}(1)M_{0,1}(2) + M_{1,0}(2)M_{0,1}(1)\right).
\end{aligned} \tag{7}$$

The covariance in the number of stage-1 and stage-2 taxa in a cluster conditional on the MRCA being in stage 1 or 2 is

$$\mathrm{Cov}(X_1, X_2; 1) = \frac{S_1}{A_1} - M_{1,0}(1)M_{0,1}(1), \tag{8}$$

$$\mathrm{Cov}(X_1, X_2; 2) = \frac{S_2}{A_2} - M_{1,0}(2)M_{0,1}(2). \tag{9}$$

And the Pearson correlation between the number of stage-1 and stage-2 in a cluster (given a stage-1 MRCA) is

$$\rho(1) = \frac{\mathrm{Cov}(X_1, X_2; 1)}{\sqrt{\mathrm{Var}(X_1; 1)\mathrm{Var}(X_2; 1)}}. \tag{10}$$

B Coalescent simulations

For simulating coalescent trees, the following parameters were sampled from a multivariate uniform distribution:

Parameter	Minimum	Maximum
β_1	0.24×10^{-3}	6×10^{-3}
β_2	0.24×10^{-4}	1.2×10^{-3}
N	10^3	5×10^4

References

1. Volz E, Pond S, Ward M, Leigh Brown A, Frost S (2009) Phylodynamics of Infectious Disease Epidemics. Genetics .

2. Frost S, Volz E (2010) Viral phylodynamics and the search for an effective number of infections. Philos Trans R Soc Lond B Biol Sci 365: 1879.

3. Volz E (2012) Complex population dynamics and the coalescent under neutrality. Genetics 190: 187-201.
